# Complete Genome and Plasmid Sequences of Raphidiopsis curvispora Strain GIHE-G1, Isolated from a Drinking Water Reservoir in South Korea

**DOI:** 10.1128/MRA.01060-20

**Published:** 2020-11-19

**Authors:** Ju-Yong Jeong, Mi-Ra Yun, Seung-Eun Oh, Seon-Min Hwang, Chan-Won Hwang, Tae-Hwa Kim, Seo-hyun Kim, Keugtae Kim

**Affiliations:** a Department of Water Environment Research, Gyeonggi-do Institute of Health and Environment, Suwon, South Korea; b Department of Environmental & Energy Engineering, Suwon University, Hwaseong, South Korea; University of Maryland School of Medicine

## Abstract

The complete genome and plasmid sequences of Raphidiopsis curvispora strain GIHE-G1, a coiled filamentous heterocyst-forming cyanobacterium isolated from a drinking water reservoir in South Korea, are reported here. The genome information is expected to improve understanding of this species.

## Abstract (precis)

The complete genome and plasmid sequences of Raphidiopsis curvispora strain GIHE-G1, a coiled filamentous heterocyst-forming cyanobacterium isolated from a drinking water reservoir in Korea, are reported here. The genome information is expected to improve understanding of this species.

## ANNOUNCEMENT

Raphidiopsis curvispora is a coiled filamentous nitrogen-fixing cyanobacterium that is morphologically and genetically similar to *R. raciborskii*. This species was first detected in a Japanese reservoir in 1995 (1) and has been reported in only a few countries around the world, including Sri Lanka, Senegal, Zambia, and Botswana (2, 3).

Surface water from the Gwanggyo Reservoir, located in Suwon, South Korea, was collected using the grab sampling method (4), and strain GIHE-G1 was identified as *R. curvispora* by determining the microscopic morphology (5). The strain was isolated using a micropipette under a microscope (6) and cultured in Jaworski’s medium (7) at 25°C with a 16:8-h light/dark cycle, according to the isolation method described in an earlier study (8). Genomic DNA (gDNA) was extracted using the DNeasy PowerSoil kit (Qiagen, USA) and sequenced using the PacBio RS II platform and the Illumina HiSeq 2500 platform. For PacBio single-molecule real-time (SMRT) sequencing, the gDNA was sheared with g-TUBEs (Covaris, USA), and size selection was performed using a BluePippin instrument (Sage Scientific). The sequencing libraries were prepared using the DNA template prep kit v3.0 for the PacBio RS II and the TruSeq DNA-free kit for Illumina, respectively, according to the manufacturers’ protocols. From the PacBio analysis, 164,020 subreads (mean subread length, 8,956 bp; *N*_50_, 12,542 bp) were generated. *De novo* assembly was performed through a three-step process (preassembly, correcting and filtering reads, constructing contigs) using HGAP v3.0 (9). Altogether, 12,280,106 raw reads were retrieved from the Illumina sequencing (GC content, 42.7%; depth of coverage, 178×). After quality control of the sequence reads and trimming of adapters using FastQC v0.11.5 and Trimmomatic v0.36, respectively, 9,227,732 reads (99.2% of reads; quality value, >30) were obtained. After *de novo* assembly, the quality-filtered sequence reads from the Illumina sequencing where 90% of bases had a Phred score of 30 or higher were applied to construct contigs more accurately using Pilon v1.21 (10).

UGENE v1.32.0 was used to construct a self-similarity dot plot to check the circularity of the contigs. The overlapping ends were trimmed. Two circular contigs (contig 1: 3,910,736 bp; depth, 203×; contig 2: 146,474 bp; depth, 269×) were retrieved. The *N*_50_ value of the contigs was 3,910,736 bp after the error correction assembly. One contig represented the chromosome, and the other was expected to represent the plasmid in a BLASTN v2.7.1+ search of the NCBI nucleotide (nt) database (11) with a threshold of 1.0E-5. The genome was annotated using NCBI PGAP v4.12 (12). Default parameters were used for all software unless otherwise noted.

The information provided by PGAP showed that the genome contained 4,057,210 bp with a 40.1% GC content, 3,566 coding sequences (CDS), and 42 tRNA, 9 rRNA, and 4 noncoding RNA (ncRNA) sequences. Eleven CRISPR arrays were also detected via PGAP. *R. curvispora* is known to be potentially toxic (13), but no toxin genes were identified in the genome.

### Data availability.

The complete genome and plasmid sequences of *R. curvispora* GIHE-G1 have been deposited in NCBI under the accession numbers CP060822 and CP060823 (GenBank), PRJNA616171 (BioProject), SAMN15834505 (BioSample), and SRR12567980 (SRA).
